# Commentary on Vorobjov et al., "Comparison of injection drug users who obtain syringes from pharmacies and syringe exchange programs in Tallinn, Estonia"

**DOI:** 10.1186/1477-7517-6-33

**Published:** 2009-11-27

**Authors:** Daniel Werb, Evan Wood

**Affiliations:** 1 British Columbia Centre for Excellence in HIV/AIDS, Vancouver, Canada; 2 School of Population and Public Health, University of British Columbia, Vancouver, Canada

## Abstract

Recent data suggest that globally, between 5% and 10% of all new HIV cases are the result of unsafe injecting practices, and experts agree that reducing these practices is key to tackling the spread of HIV. And yet, despite the overwhelming evidence that providing sterile syringes to injection drug users (IDU) through syringe exchange programs (SEPs) or other means is an effective way of reducing HIV transmission among high-risk subpopulations, IDU in most settings still do not have access to sterile injecting equipment or if they do, access remains too restricted to effectively reduce the risk of HIV transmission. Vorobjov and colleagues have presented in this journal an interesting and timely study from Estonia comparing individuals who obtain syringes from SEPs and those who obtain syringes from pharmacies. As the authors point out, Estonia faces an unacceptably high HIV incidence rate of 50 new HIV cases per 100,000, this rate driven primarily by injection drug use. As such, the authors argue that Estonia's SEP network does not have the capacity to serve a growing IDU population at risk of transmitting HIV and pharmacy dispensation of clean syringes may be one potential approach to decreasing syringe sharing among high-risk injectors. It may be overly optimistic to consider the impact of higher threshold interventions such as pharmacy-based SEPs, given that IDU populations that engage in HIV risk behaviours such as syringe sharing are often hidden or hard to reach. Despite the need for a cautious approach, however, the findings presented by Vorobjov et al. may chart one potential course towards a more comprehensive societal response to reducing the health harms associated with injection drug use.

## 

A global consensus has been reached regarding the primary role of syringe sharing in driving the HIV epidemic among injection drug users (IDU), and there is growing international recognition of the interventions required to address this public health crisis. Recent data suggest that globally, between 5% and 10% of all new HIV cases are the result of unsafe injecting practices [1,2], and experts agree that reducing these practices is key to tackling the spread of HIV. Despite this scientific consensus, there still exists a shortage of resources allocated towards the scale-up of interventions to address the harms associated with syringe sharing. For instance, while approximately 83% of all countries reporting HIV-infection among IDU subpopulations have at least one syringe exchange program (SEP), certain regions such as Eastern Europe and the former Soviet Union continue to report that 70% to 90% of all HIV infections are the result of injection drug use [2]. This problem persists despite the fact that the World Health Organization and a number of other multilateral organizations and national public health authorities have endorsed SEPs as a simple method of reducing the risks for HIV transmission associated with syringe sharing [1]. And yet, despite the overwhelming evidence that providing sterile syringes to IDU through SEPs or other means is an effective way of reducing HIV transmission among high-risk subpopulations [3], IDU in most settings still do not have access to sterile injecting equipment or if they do, access remains too restricted to effectively reduce the risk of HIV transmission [4].

This is of concern, particularly in light of the many interventions available to policymakers considering how best to distribute sterile syringes to different IDU subpopulations. For example, Riley and colleagues found that first-time syringe exchange participants who acquired sterile syringes from mobile sites (i.e., syringe exchange vans) in Baltimore were more likely to be frequent injectors, suggesting that these interventions may effectively target highly dependent individuals [5]. Other researchers have confirmed that, while IDU accessing SEPs often have higher risk profiles for the transmission of HIV and other blood-borne diseases, SEPs themselves are an efficient means of reducing health risks among hard-to-reach, stigmatized, and hidden populations [6,7].

Vorobjov and colleagues have presented in this journal an interesting and timely study from Estonia comparing individuals who obtain syringes from SEPs and those who obtain syringes from pharmacies [8]. As the authors point out, Estonia faces an unacceptably high HIV incidence rate of 50 new HIV cases per 100,000, this rate driven primarily by injection drug use. As such, the authors argue that Estonia's SEP network does not have the capacity to serve a growing IDU population at risk of transmitting HIV. Within this context, the study investigated whether pharmacies that dispensed sterile injecting equipment complement SEPs in serving the needs of Estonian IDU, and whether differences exist between individuals that primarily use SEPs compared with those that primarily use pharmacies to acquire sterile injecting equipment.

In their analysis, Vorobjov and colleagues found that individuals who reported using pharmacies as their primary source of sterile equipment exhibited lower risks of HIV transmission than IDU who primarily used SEPs. Like many studies of IDU, this study is limited by its cross-sectional design and its use of non-random sampling. As well, the possibility exists that the merging of users of SEPs and pharmacies within the sample may have led to unmeasured confounders. Nevertheless, it presents important new data and highlights the potential of pharmacies in playing an early preventive role in limiting the risk trajectory of IDU, and it does so in a region that is hard hit by an injection-fuelled HIV epidemic and is in need of epidemiologic research. If, as the authors suggest, IDU who visit pharmacies represent a subpopulation at an earlier stage of their injecting careers, pharmacies could play a key role in connecting IDU to treatment such as methadone and preventive services prior to a transition to riskier use of injection drugs [9]. If feasible, pharmacies could also be potential locales for the provision of addiction treatment, counseling, detoxification referral, or social services. Further longitudinal research in this area is therefore needed.

The limits of SEPs in servicing IDU are well-known [10]. As such, it may be overly optimistic to consider the impact of higher threshold interventions such as pharmacy-based SEPs. Despite the need for a cautious approach, however, the findings presented by Vorobjov et al. may chart one potential course towards a more comprehensive societal response to reducing the health harms associated with injection drug use, and could also hold lessons for policymakers and health authorities in other settings that may be struggling to reduce the transmission of HIV among hard to reach IDU populations.
